# Improving nursing education curriculum as a tool for strengthening the nurse–client relationships in maternal and child healthcare: Insights from a human-centered design study in rural Tanzania

**DOI:** 10.3389/fpubh.2023.1072721

**Published:** 2023-02-03

**Authors:** Kahabi Ganka Isangula, Eunice Siaity Pallangyo, Eunice Ndirangu-Mugo

**Affiliations:** ^1^School of Nursing and Midwifery, Aga Khan University, Dar es Salaam, Tanzania; ^2^School of Nursing and Midwifery, Aga Khan University, Nairobi, Kenya

**Keywords:** nursing, human-centered design, maternal and child health, curriculum, Tanzania, rural, Africa

## Abstract

**Background:**

There are growing evidence of poor nurse–client relationships in maternal and child health (MCH). The nursing curriculum forms an important entry point for strengthening such relationships, consequently improving client satisfaction with nurses' competencies, confidence in the formal healthcare system, healthcare-seeking practices, continuity with care, and MCH outcomes.

**Objective:**

MCH nurses and clients were invited to design an intervention package (prototype) to improve nurse–client relationships using a human-centered design (HCD) approach.

**Methods:**

A multi-step HCD approach was employed to first examine the contributors of poor nurse–client relationships using nine focus group discussions with nurses and clients and 12 key informant interviews with MCH administrators. Then, three meetings were held with 10 nurses, 10 clients, and 10 administrators to co-develop an intervention package to address the identified contributors. The solutions were validated by collecting qualitative information through six focus groups with nurses and MCH clients who were not involved in the initial HCD stages. Finally, refinement and adaptation meetings were held with 15 nurses, 15 clients, and 10 administrators. The data were managed with NVivo 12 software and analyzed thematically.

**Results:**

Nursing curriculum challenges contributing to poor nurse–client relationships in MCH care included inadequate content on nurse–client relationships specifically topics of customer care, communication skills, and patient-centered care; an inadequate practice on communication skills within nursing schools; and the absence of specific trainers on interpersonal relationships. Consequently, improving the nursing curriculum was one of the interventions proposed during the co-design and rated by participants as highly acceptable during validation and refinement meetings. Suggested improvements to the curriculum included increasing hours and credits on communication skills and patient-centered care, including customer care courses in the curriculum and creating a friendly learning environment for clinical practice on strengthening interpersonal relationships.

**Conclusion:**

Improving the nursing curriculum was considered by nurses and clients as one of the acceptable interventions to strengthen nurse–client relations in MCH care in rural Tanzania. Nursing education policy and curriculum developers need to ensure the curriculum facilitates the development of much-needed interpersonal skills among nursing graduates for them to have positive therapeutic interactions with their clients.

## 1. Introduction

There is a broad consensus that nurses form a critical component of the human resource for the health workforce that is charged with the delivery of maternal and child health (MCH) services globally. Nurses have a unique role in MCH service delivery within primary healthcare settings as they monitor pregnancy, perform deliveries, offer postnatal care and family planning services, and provide public health education worldwide (1–4). In sub-Saharan Africa (SSA), nurses are the reliable source of medical and public health information and counseling on a range of health issues, particularly on the care of women, newborns, and under-five children (2–4). Despite the critical role of nurses, there is growing evidence of client dissatisfaction with providers' competencies in MCH care in recent years (5–13). Client dissatisfaction appears to be centered around incompetence related to nurses' technical skills in the delivery of MCH care; their reliability, assurance, and confidentiality; and inadequate patient engagement in making MCH care decisions. There is also overwhelming client dissatisfaction with nurses' behaviors related to their professional conduct, attitudes, communication, and language as well as the violation of some of the client's rights in Tanzania and other African countries (6–11). Such dissatisfaction continues to not only obscure the positive contribution of nurses in MCH care and public health but also contribute to a negative impact on client confidence in formal healthcare systems, poor healthcare seeking practices, and discontinuity with care, which is partly indicated by persistently high home deliveries and poor MCH outcomes in Tanzania and other settings (10–17).

There have been notable efforts to address client dissatisfaction with providers including nurses in different settings. Healthcare governance tools such as complaints mechanisms, policies, guidelines, client service charters, governance committees, and nursing professional boards have been considered within and outside Tanzania (17–20). So far, there has been no reliable evidence of their effectiveness. Similarly, there have been efforts to implement interventions focusing on both providers and clients. Training for providers on communication skills and competencies and essential skills in the delivery of patient-centered care on the one hand and enhancing clients' literateness, information-seeking capacity, participation in care, and questioning skills, on the other hand, have been implemented with unclear results (17–20). The problem with these efforts, however, is that they have failed to consider the complexities of provider–client relationships within MCH care. Evidence on therapeutic relationships from rural Tanzania indicates that provider–clients relationship is complex and interventions to strengthen such relationships may be impacted not only by patients' socioeconomic status, literacy, and behaviors but also by provider interpersonal skills and health system challenges (17–22). A key health system challenge that is more likely to negatively impact nurse–client relationships is the quality of graduates of nursing training institutions. Evidently, the nursing curriculum forms an important entry point for strengthening such relationships consequently improving client satisfaction with nurses' competencies, confidence in the formal healthcare system, healthcare-seeking practices, continuity with care, and MCH outcomes. Equipping nursing students with adequate verbal and non-verbal interpersonal communication and customer care skills is critical in strengthening nurse–client relationships and this can commence with the nursing education curriculum (21, 22). However, researchers have not treated the contribution of nursing curriculum on nurse–client relationships in much detail.

Between January and September 2022, the Aga Khan University (AKU) in Tanzania implemented a human-centered design (HCD) intervention in the rural region of Shinyanga. Nurses and clients from MCH clinics were invited to collaborate with the research team for the co-development of an intervention package (prototype) for their therapeutic relationships. HCD has been described as an innovative approach applied to solving complex problems by leveraging the insights and experiences of end users to co-design solutions that may be prototyped and refined iteratively (23–28). The interventions designed through the HCD process have been documented to be more successful and sustainable in comparison to the traditional approaches for solving problems within the healthcare sector (27). This paper examines the findings indicating that improving the nursing education curriculum may be an important tool for strengthening the nurse–client relationships in MCH care in rural settings. The evidence generated is expected to guide nursing education policy and curriculum developers in restructuring the curriculum to address the much-needed interpersonal skills gaps among nursing graduates to fuel positive therapeutic interactions with their clients.

## 2. Methods

### 2.1. Design

A protocol for this study has been published elsewhere (29). In summary, a five-step HCD approach was employed as a framework for co-designing an intervention package and strengthening the nurse–client relationships using qualitative descriptive design using focus group discussions (FGDs), key informant interviews (KIIs), and consultative meetings. Qualitative descriptive design was regarded more appropriate for this inquiry in answering two key questions: (i) What are the contributors of poor nurse–client relationships in MCH care in rural Tanzania? and (ii) What intervention package (prototype model) for strengthening nurse–client relationships could emerge in the HCD process engaging nurses and clients in the study settings? Furthermore, a qualitative descriptive approach is more appropriate for this study as it was aimed at generating a rich understanding and describing the nurse–client relationships without testing an existing theory (30). As noted earlier, HCD is an approach to solving complex problems using a series of iterative and habitually nonlinear steps to develop solutions (23–28). In the HCD approach, beneficiaries or end users are invited to partner with the research team to design and evaluate the emerging solutions to better comprehend and solve the challenges they have identified.

### 2.2. Settings

This study was conducted in Shinyanga, one of the regions predominantly inhabited by the Bantus and located in the Lake Zone. Isangula (17) described this rural region in detail. In summary, Shinyanga is one of the low-income regions of Tanzania. The region is administratively divided into six districts: Shinyanga Municipal Council (MC), Msalala DC, Shinyanga District Council (DC), Kahama MC, Ushetu DC, and Kishapu DC. The choice of Shinyanga was because, first, it has a higher rural population with more than 95% of rural occupancy (17). Second, the ethnic population is predominantly Sukuma (Bantus) who share many sociocultural beliefs and practices with minimal differences. Due to higher rural occupancy and its near homogeneity, the region formed an ideal exemplar of other rural regions of Tanzania and Africa. Third, although there have been some efforts to improve provider–client relationships in the region, local data indicate alarming concerns about poor therapeutic relationships in MCH care within primary healthcare facilities (17). Within the Shinyanga region, we purposefully selected Shinyanga MC because MCH clients in this district have wider access to both informal (traditional care) and the formal healthcare system (mostly public and few private and faith-based facilities) (17).

### 2.3. Study population, sample size, sampling, and data collection

The five-step HCD process was employed, namely, community-driven inquiry, co-design, validation, refinement, and documentation and sharing of lessons learned. We used a combination of qualitative research methodologies to explore community and individual perspectives of the drivers of poor nurse–client relationships during the community-driven inquiry step. Approximately nine FGDs and 12 KIIs were conducted with purposefully selected nurses and midwives, women attending MCH services, and MCH stakeholders, using a semistructured interview guide in the Swahili language. Nurses and clients were recruited through MCH managers, clients through their facilities of MCH care, and administrators invited after obtaining their phone numbers from the district registry. All interviews were conducted at a convenient location. The location was confirmed with the participants in advance to enable them to identify a suitable alternative if required. Upon arrival at the predetermined interview venue, research assistants offered information about the study, requested and obtained informed consent, and engaged participants in a semistructured audio-taped discussion lasting for approximately 45–60 min. The findings of community-driven inquiry informed the co-design step in which a transdisciplinary team purposively selected MCH nurses and clients from those who participated in the first step, five administrators, and other five relevant stakeholders (30 members). The team gathered for 3 days, identified key contributors based on discovery findings, and designed an intervention package (rough prototype) with the highest potential to improve nurse–client relationships. We employed a group-based consensus-building approach to discuss the factors and generate potential interventions. We further used a group-based rating of the emerging interventions considering their acceptability and feasibility. Four co-design groups rated the emerging interventions using a score of 0–10 for both feasibility and acceptability. Scores from each group were summarized and a consensus was reached in a broader group on the interventions with higher scores that formed a rough prototype, The findings of codesign meetings informed step 3 of the HCD process, which involved gathering qualitative insights on the rough prototype in Shinyanga MC. The aim was to gather nurses' and clients' feedback on the rough prototype using FGDs (six sessions), with a new group of purposively sampled respondents who were not part of the initial two HCD steps to identify features appealing to both nurses and clients for strengthening their therapeutic relationship as a pathway for increasing MCH service satisfaction, uptake, and continuity. The recruitment and interview process for the insight-gathering inquiry was similar to what is described for discovery inquiry (as described earlier). The findings of the insight-gathering step informed the refinement step. During this step, the design team reconvened for 2 days to evaluate the feedback on the rough prototype as well as refine and adapt the prototype. Approximately 10 representatives of insight-gathering inquiry were selected by their peers to join the 30 participants of co-design meetings in the refinement and adaptation process (40 members). The refinement meeting resulted in the final prototype model. The research team synthesized the lessons learned and are currently being shared through local and international forums. This paper forms part of the documentation and sharing of the evidence generated.

We recruited three research assistants with Diplomas in health sciences and trained them on the HCD process and techniques pertaining to this study. The discussion, interview, and consultative meeting guides were developed collaboratively, pretested in purposefully selected settings, and refined to enhance readiness for use in the actual data collection process. The PI maintained close and supportive supervision of research assistants throughout the data collection and analysis stages to maximize data quality.

### 2.4. Data management and analysis

The HCD steps generated a wealthy amount of data from FGDs, KIIs, and consultative meetings. Data transcription and translation occurred simultaneously by research assistants and were verified by the research team. Interview transcripts were deidentified, pseudonyms were generated for each participant, and data were uploaded into NVivo 12 software (QSR International) for management and thematic coding. We employed a stepwise approach for a deductive thematic analysis of the interview transcripts (31). The first step involved the examination of the research questions by the research team and consensually generated several themes. This resulted in an analytical matrix of the main themes and subthemes. The second step involved exporting the individual transcripts and phrases (codes) representing participants' responses to investigators' questions to relevant themes and related subthemes within NVivo. Again, the research team used a consensus-building used approach to decide on the inclusion of codes that did not fit in the pre-developed subthemes and themes; the codes were excluded when they did not provide critical value to the study, as confirmed by subjective and objective evaluations. The final step was exporting coded data within NVivo to Microsoft Word (Microsoft Corporation) for interpretative analysis and report generation.

### 2.5. Ethics approval

This ethics clearance for this study was obtained from the AKU Ethics Review Committee and the National Institute for Medical Research (NIMR/HQ/R.8a/Vol. IX/3906). Written approvals to conduct the study were also obtained from the Regional Medical Officer of Shinyanga and the Municipal Medical Officer in Shinyanga. Similarly, verbal approvals were obtained from the managers of the selected healthcare facilities from where nurses and clients were recruited after providing letters from the region and district medical officers and copies of ethical clearance. The research team ensured the responsible conduct of research by obtaining verbal consent from all participants before participation and recording it as part of the interview transcript.

## 3. Results

### 3.1. Participant demographics

The community-driven discovery inquiry involved 30 nurses (four FGDs), 36 clients (five FGDs), and 12 stakeholders (MCH administrators and a representative of the Health Facility Governance Committee). Co-design meetings involved an equal number (10) of nurses, clients, and stakeholders to ensure representativeness. The validation inquiry involved 22 nurses (three FGDs) and 26 clients (three FGDs). Refinement meetings involved 15 nurses, 15 clients, and 10 stakeholders. Females accounted majority of participants: 90% discovery, 90% co-design, 96% validation, and 90% refinement participants. On the one hand, most nurses had a higher level of education level (those with college and above were 77% of discovery, 90% co-design, 100% validation, and 85% refinement participants) as compared to clients (those with secondary and below were 86% discovery, 70% co-design, 100% validation, and 90% refinement participants) (Table 1).

**Table 1:** Participants' demographics.

	**Community driven-discovery**				**Co-design meetings**				**Validation inquiry**			**Refinement meetings**			
**Category**	**Nurses (%)**	**Clients (%)**	**Admin (%)**	**Total (%)**	**Nurse s(%)**	**Clients (%)**	**Admin (%)**	**Total (%)**	**Nurses (%)**	**Client s(%)**	**Total (%)**	**Nurses (%)**	**Clients (%)**	**Admin (%)**	**Total(%)**
	***n** =* **30**	***n** =* **36**	***n** =* **12**	***N** =* **78**	***n** =* **10**	***n** =* **10**	***n** =* **10**	***N** =* **30**	***n** =* **22**	***n** =* **26**	***N** =* **48**	***n** =* **15**	***n** =* **15**	***n** =* **10**	***n** =* **40**
Gender															
Female	26(87)	36	8 (67)	70(90)	10	10	7(70)	27(90)	20(91)	26(100)	46(96)	13 (87)	14(93)	8(80)	35 (87.5)
Male	4 (13)	0	4 (33)	8(10)	0	0	3 (30)	3(10)	2(9)	0	2(4)	2(13)	1(7)	2(20)	5 (12.5)
Age															
< 30	6 (20)	22(61)	0	28(36)	1(10)	4(40)	0	5(17)	5(23)	20(77)	25(52)	7(47)	3(20)	0	10 (25)
31–40	14(46)	13(36)	3(25)	30(38)	5 (50)	6(60)	1(10)	12(40)	6(27)	4(15)	12(25)	6(40)	8(53)	1(10)	15(37.5)
41–50	5(17)	0	8(67)	13(17)	3(30)	0	7(10)	10(33)	6(27)	1(4)	7(15)	2(13)	4(27)	8(80)	14(35)
>50	5(17)	1(3)	1(8)	7(9)	1(10)	0	2(20)	3(10)	5(23)	1(4)	4(8)	0	0	1(10)	1(2.5)
Education															
None	0	5(14)	0	5(6)	0	1(10)	0	1(3)	0	0	0	0	3(20)	0	3(7.5)
Primary	1(3)	17(47)	0	18(23)	0	3(30)	0	3(10)	0	16(62)	16(34)	1(7)	9(60)	0	10(25)
Secondary	6(20)	9(25)	1(8)	16(21)	1(10)	3(30)	1(10)	5(17)	0	10(38)	10(21)	5(33)	2(13)	0	7(17.5)
College	21(70)	4(11)	2(17)	27(35)	6(60)	2(20)	4(40)	12(40)	21(95)	0	21(44)	8(53)	1(7)	8(80)	17(42.5)
University	2(7)	1(3)	9(95)	12(15)	3(30)	1(10)	5(50)	9(30)	1(5)	0	1(2)	1(7)	0	2(20)	3 (7.5)
Years of MCH work/leadership (nurses & administrator)															
< 2	4(13)	NA	1(8)	NA	1(10)	NA	2(20)	NA	6(27)	NA	NA	NA	NA	2(20)	2(20)
2–4	20 {67)		2(17)		7(60)		6(60)		9(41)					4(40)	4(40)
>5	6(20)		9(95)		2(20)		2(20)		7(32)					4(40)	4(40)

### 3.2. Findings from the community-driven discovery inquiry

The findings of community-driven inquiry have been published elsewhere (19). A range of nursing curriculum challenges contributing to poor nurse–client relationships in MCH care emerged. Some participants linked poor nurse–client relationships to inadequate coverage of interpersonal relationship content in the nursing curriculum. Topics such as customer care, communication skills, and centered patient-centered care were considered inadequate in the existing curriculum. Some participants considered inadequate practice on communication skills within nursing schools and the absence of specific trainers on interpersonal relationships as contributing to poor nurse–client relationships among graduates. Some considered the reduction of years of nursing diploma studies from 4 years (previously) to 3 years to have contributed to inadequate content and duration for practical skills in interpersonal relationships. Some participants commented:

*Graduates of nursing schools nowadays have very limited communication and customer care skills. You find a recently hired nurse has bad language and poor client reception. This is because communication skills and customer care topics do not have enough hours in the nursing curriculum (Nurse, Hospital)*.

*I hear that they used to study nursing for 4 years but now it is only 3 years. This means the content on nurse and client relationships and time for clinical practice on these skills has been cut short and I think that is why many nurses have poor customer care (Client, Dispensary)*.

### 3.3. Findings from consultative co-design meetings

Synthesis meetings formed the first series of co-design meetings. Community-driven inquiry findings were presented, and participants examined the findings building on personal experiences, insights, and questions to generate a comprehensive understanding of the challenges of nurse–client relationships in Shinyanga. The results of the synthesis meeting indicated a broad consensus on the contributors of poor nurse–client relationships with some addition of the contributors. For instance, a few participants went ahead to link poor nurse–client relationships to an inadequate screening of nursing students during enrollment resulting in the enrollment of students with limited nursing ethics (as mentioned later). The ideation meeting involved group discussion to brainstorm to generate the “how might we” questions. This facilitated the generation of 82 ideas on how to improve nurse–client relationships each with several activities. A prototype and co-creation meeting brought together participants in three groups to evaluate the ideas generated during the ideation meeting and the emerging categories considering pros, cons, and feasibility. The ideas for strengthening nurse–client relationships through curriculum improvements included increasing hours and credits on communication skills and patient-centered care, the inclusion of customer care courses in the curriculum, and creating a friendly learning environment for clinical practice on strengthening interpersonal relationships. Furthermore, one administrator recommended reverting to the old system of screening where only highly motivated students were enrolled in nursing schools. One participant commented:

*We need to go back to a system that was used previously to screen students starting with looking at different dimensions to ensure that only those who are motivated to become nurses are enrolled. Nowadays they just enroll anyone who graduates from secondary schools without screening for those who are self-motivated. Maybe because employment in the nursing sector is easy. They need to go back to the old system of screening students (MCH administrator)*.

The 82 ideas generated were further grouped into 24 categories considering conceptual convergence and similarities between them. Through consensus building, participants were divided into four groups and rated the 24 categories (and their related activities) considering feasibility (0–10 scores) and acceptability (0–10 scores) among nurses and clients. The total scores ranged from 58 out of 80 for disciplinary measures for abusive nurses and clients (highest), followed by 56 out of 80 for awards and recognition for nurses, 52 out of 80 for strengthening complaints mechanisms followed by 49.5 out of 70 for improving nursing school curriculum, 49.5 out of 80 for ensuring the availability of resources, 49 out of 80 for developing nursing leaders, 48 out of 80 for the promotion of patient-centered care, and 32.5 out of 80 for ensuring the availability of mental health services and support for nurses and clients (lowest). The meeting resolved to consider the seven interventions with the highest scores, including (i) disciplinary measures for abusive nurses and clients; (ii) awards and recognition of good nurses; (iii) strengthening complaints mechanisms; (iv) improving nursing curriculum; (v) improving availability of resources; (vi) improving the efficiency of nursing leaders; and (vii) provision of patient-centered care. This indicated that improvement of the nursing school curriculum was the fourth highest rated intervention forming part of the ‘rough prototype model' that was then subjected to a validation step.

### 3.4. Findings from the validation/insight gathering inquiry

During FGDs with nurses and clients, improving the nursing curriculum was reaffirmed as one of the key interventions for strengthening nurse–client relationships. Most participants supported the ideas proposed for improving nurse–client relationships in the co-design meetings (as mentioned earlier). Some participants recommended the Ministry of Health to monitor and build the capacity of instructors in nursing schools for them to be able to train their students on nurse–client relationships. Some recommended specific programs to orient newly employed nurses on nurse–client relationships possibly recognizing that they may have missed the opportunity to acquire these skills in nursing schools. Some participants commented:

*The Ministry of Health needs to visit nursing institutions to monitor the provision of education on interpersonal relationships and train the instructors [on nurse-client relationships] so them to be able to train their nursing students on how to better care for their patients [Client, Dispensary]*.

*There is a need to have a special induction course on nurse-client relationships for newly recruited nurses because this allows them to understand the actual situation which may be different from what they learnt in a classroom at nursing schools. They need to be trained on how to establish and maintain good relationships with their clients [MCH administrator]*.

The insights gathered through FGDs by interviewing the nurses and clients who were not part of the initial HCD steps indicated a broad consensus that the seven interventions are more likely to improve nurse–client relationships. A range of benefits and disadvantages of these interventions were cited. Of note, the benefits of these interventions cited by participants of validation inquiry largely focused on nurses and clients. On the one hand, these interventions were considered to increase nurses' commitment, confidence, and morale, increase closeness and partnership between nurses and clients and improve clients' health-seeking behaviors, continuity with care, participation in care, and adherence to nurses' instructions consequently improving MCH outcomes. The disadvantages of these interventions included fears among some participants that some interventions require more time and resources therefore may be less feasible as compared to other interventions.

### 3.5. Findings from the prototype refinement/adaptation meeting

The findings of the refinement and adaptation meetings indicated a consensus that all the interventions proposed were considered acceptable. However, there were some concerns about the feasibility of the curriculum-related intervention. Although restructuring the nursing curriculum as a tool for strengthening the nurse–client relationship was rated by all groups as highly acceptable (38 out of 40), it was rated less feasible by all groups (8 out of 40) considering the study contexts. This is because of the time and multistakeholder efforts needed for the successful improvement of the nursing curriculum. Consequently, curriculum improvement was rated seventh and the final prototype model with four interventions included: (i) patient-centered care; (ii) awards and recognition for good nurses; (iii) improving complaints mechanisms; and (iv) simple disciplinary measures for bad nurses. However, curriculum improvement was considered a worthy endeavor to pursue alongside other interventions. One administrator commented:

*Changing the curriculum will take a very long time because these documents cannot change overnight. However, we need to continue advocating for curriculum improvement because it is one of the important strategies for improving nurse-client relationships (MCH Administrator)*.

### 3.6. Documentation and sharing the lessons learned

The research team embraced a number of strategies to disseminate the findings of this study pilot. We first deposited the emerging publications in AKU networks including eCommons as well as presented the findings in institutional forums. We are currently sharing results with local nursing and healthcare authorities by sending summary reports to the district and regional medical officers, nursing and midwifery councils, the Ministry of Health, and the National Institute for Medical Research for dissemination through government channels. Finally, we are sharing the results of the intervention study through peer-review journals and at international conferences.

## 4. Discussion

This study was conducted to co-design of an intervention package for strengthening nurse–client relationships in MCH care in rural Tanzania using the HCD approach. The research team partnered with nurses, clients, and other MCH stakeholders in the Shinyanga region in a series of HCD steps to co-develop an intervention package. As noted in the study protocol (29), the choice of Shinyanga was partly because of the homogeneity of the local population with limited socio-cultural variations and the evidence from previous performance reports and research that indicate persistent challenges of poor patient–provider relationships in the region (17, 22). This means a focus on Shinyanga embraced the need for reinforcing our understanding of the unique challenges that nurses and clients continue to face in MCH care and public health in this setting so as to develop a context-specific intervention that may be applicable within the region and in a similar context. This was in keeping with the recommendations of the intervention evaluation and development framework proposed by the UK Medical Research Council, which emphasize the need to consider contexts throughout the adaptation and implementation of interventions (29). Therefore, the findings emerging in Shinyanga may serve as an exemplary model for further testing of curriculum improvement activities in other parts of Tanzania and Africa. Furthermore, the focus on developing an intervention package through the HCD process was because of the wide recognition of how strong nurse–client relationships have been documented to have overarching results in public health promotion and healthcare. Strong nurse–client relationships have been linked to improved quality of care, improved partnership in healthcare decisions, improved adherence to instructions and medical interventions, and improved health outcomes (13, 18, 32, 33). Therefore, acceptable interventions co-designed by nurses and clients themselves provide an opportunity for embracing the nurse–client relationship as a tool for addressing some of the challenges of MCH care and public health.

The first step of the HCD process unmasked a range of nurses, clients, and institutional contributors to poor nurse–client relationships (22). Nurse contributors to poor nurse–client relationships included a range of curriculum-related challenges that were said to contribute to poor nurse–client relationships. Poor customer care and communication skills among nurses emerged as contributing to poor nurse–client relationships and they were cited to result from limited content on topics related to interpersonal relationships between nurses and clients, particularly customer care, communication skills, and patient-centered care in the nursing curriculum. There were also widespread concerns about limited clinical practices on communication skills and the absence of instructors with sufficient expertise in interpersonal relationships in therapeutic settings. To partly address the nurse contributors to poor nurse–client relationships, a suggestion was made for improving the nursing curriculum as a strategy for generating nursing graduates with self-drive and good relationships with clients (22). Specific activities for improving the curriculum were proposed by nurses, clients, and administrators including increasing content and extending the duration of communication skills and customer care courses to the nursing curriculum. Previous literature has highlighted concerns about limited contents of communication and customer care skills in the nursing curriculum without linking it to poor nurse–client relationships (13, 18, 20, 21, 34). Some literature has identified communication skills and customer care as the key ingredients of strong nurse–client relationships without acknowledging that the nursing curriculum provides an opportunity for learning such skills (35–38). However, a recent review of the literature on SSA has recommended the inclusion of communication skills in the nursing curriculum as a key strategy for improving nurse–client relationships (20). This implies that there is a need for continued advocacy on curriculum improvement as a tool for strengthening nurse–client relationships in MCH care and public health promotion.

The second step of the HCD process involved co-design meetings, the contributors of poor nurse–client relationships, and suggestions offered during community-based inquiry were examined closely and about 82 ideas were proposed. Rating of these ideas resulted in a rough prototype model with seven interventions which included simple disciplinary measures, awards, and recognition of good nurses, complaints mechanisms, enhancing the availability of resources, improving the efficiency of leaders, and patient-centered care. Improving the nursing curriculum was rated fourth in the rough prototype model. At this stage, the need for improving the nursing curriculum was rated as highly acceptable. Although there was recognition that improving the curriculum may be less feasible due to the time and resources required, an emphasis was made that it is one of the important interventions to consider explaining why it formed part of the rough prototype model.

The third step of the HCD process involved validation of the rough prototype model using focus group discussions with a new group of nurses and clients. The need for adding customer care courses and extending communication skills courses to the nursing curriculum was reaffirmed at this stage. It was further suggested that there is a need for increasing hours and credits on communication skills, customer care and patient-centered care, and creating a friendly learning environment for clinical practice on strengthening interpersonal relationships. It is important to note that poor communication skills and customer care have not only been documented as key drivers of poor provider–client relationships but also building these skills has been recommended as a key strategy for improving such relationships (6, 10–20).

Although it is widely recognized that nursing education curricula need to be adapted and frequently reviewed to accommodate the changing needs, healthcare environment, and service delivery practices (35–39), adding credits and hours for topics related to strengthening nurse–client relationships has not been strongly regarded as one of the adaptations that need to be made. However, the need for improvement of the clinical learning environment has always been a recommendation of most research examining nursing curriculums with very few linking this to strengthening nurse–client relationships (37–39). For instance, a self-assessment study of nursing students conducted by Suikkala et al. (40) recommended that maintenance of a better clinical learning environment and teaching approaches are needed to ensure that students acquire the necessary skills for strengthening nurse–client relationships. This indicates that nursing curriculum improvement forms not only an entry point for improving technical competence among nursing graduates but also could facilitate effective learning of essential skills needed for positive therapeutic interactions with their clients.

The fourth step of the HCD process involved refinement meetings. The insights and suggestions of nurses who participated in the validation of the rough prototype model were reviewed and discussed. A fresh rating of the interventions of the rough prototype resulted in the final prototype model with four interventions including patient-centered care, awards and recognition for nurses, complaints mechanisms, and simple disciplinary measures but excluding the curriculum improvement which was rated seventh. The reason curriculum improvement was excluded from the final prototype model was that it scored less in feasibility rating because of the complexity of its implementation requiring massive resources, bureaucratic process, and extended time (41). However, there were massive calls from participants for the need to implement curriculum improvement interventions alongside the final prototype model. This explains why this article is dedicated to curriculum improvement as a tool for improving nurse–client relationships in MCH care and public health promotion.

One of the key nursing professional bodies in the country is the Tanzania Nursing and Midwifery Council (TNMC). The council's website (www.tnmc.go.tz/) indicates that TNMC is a professional regulatory authority charged with ensuring that services provided by nurses and midwives in Tanzania are of an acceptable standard and safe to their clients. On top of the accreditation of nurses and midwives in the country, the council is responsible for prescribing the standards of proficiency necessary for admission, administering nursing licensing examinations and setting standards of nursing training and education, and evaluating of quality of education. This indicates that TNMC forms an important entry point for strengthening nurse–client relationships through curriculum improvements. Building on the findings of this study, TNMC could encourage nursing institutions to review the current curriculums and increase credits hours on communication skills and patient-centered care and ensure the inclusion of customer care courses. As a regulatory body charged with accreditation of nursing education providers, setting standards for nursing education and education quality monitoring, TNMC could ensure that nursing institutions have a friendly learning environment for their students to effectively practice strategies for strengthening interpersonal relationships and learn how to address the factors shaping poor nurse–client relationships (17, 22). Relatedly, TNMC may consider giving much weight to skills related to communication, customer care, patient-centered care, and nurse–client relationships in nursing accreditation and licensing examinations. This will ensure that nursing graduates licensed by TNMC are better equipped with essential skills for positive interactions with their clients in MCH care both within nursing training institutions and through licensing examinations. It is only through this approach, the improvements in Nursing Education Curriculum could then be an important tool for strengthening the nurse–client relationships in MCH care consequently contributing to increased healthcare seeking, continuity with care, and better health outcomes among clients.

### 4.1. Limitations

The application of HCD to develop a prototype for improving nurse–client relationships may have some limitations. The HCD study in Shinyanga involved nurses as exemplars of healthcare providers to codesign a prototype for and improve provider–client relationships in MCH care in a rural setting. However, clients in MCH care interact with multiple teams of providers in healthcare settings and have previous experiences which may pre-determine how they interact with nurses. For instance, Ozawa and Walker (42) indicated that a mixture of patients' prior experiences in healthcare settings and their interactions with non-medical personnel may impact how they construct interpersonal relationships with medical providers. Therefore, if a similar study is conducted with other providers, for instance, doctors, and in a different setting and context may generate a different prototype. However, since this is the first study in this context, we suggest that future inquiries need to extend beyond the nursing profession and rural context. Furthermore, although we are emphasizing nursing curriculum improvement as a tool for improving interpersonal skills among nurses, such intervention alone may not effectively address all the contributors to poor nurse–client relationships identified during the community discovery inquiry component of the HCD study (22). We encourage practitioners and researchers to consider a range of interventions proposed by nurses and clients during the HCD study alongside efforts to improve the nursing training curriculum. These include awards and recognition for good nurses, disciplinary measures for bad nurses, continued mentorship, continued professional development, and strengthening complaints mechanisms just to cite a few.

### 4.2. Conclusion

In conclusion, the use of HCD provided an opportunity for the researchers to partner with nurses and clients in exploring the challenges of nurse–client relationships and co-development of the acceptable interventions. Improving the nursing curricula was considered by nurses and clients as one of the acceptable interventions to strengthen nurse–client relations in MCH care in rural Tanzania. Nursing education policy and curriculum developers need to ensure that existing curriculums are adapted to facilitate the development of much-needed interpersonal skills among nursing graduates for them to have positive therapeutic interactions with their clients.

## Data availability statement

The original contributions presented in the study are included in the article/supplementary material, further inquiries can be directed to the corresponding author.

## Ethics statement

The studies involving human participants were reviewed and approved by National Institute for Medical Research. The patients/participants provided their written informed consent to participate in this study.

## Author contributions

KI designed the study, solicited funding, and developed the initial draft of the manuscript. EP and EN-M participated in the project conception and design, critically reviewed the manuscript, and provided inputs for improvement. All authors contributed to the article and approved the submitted version.
